# PD-1/PD-L1 inhibitor plus chemotherapy versus bevacizumab plus chemotherapy in first-line treatment for non-squamous non-small-cell lung cancer

**DOI:** 10.1136/jitc-2021-003431

**Published:** 2021-11-08

**Authors:** Hui Yu, Ping Chen, Liangping Xia, Sha Fu, Chen Chen, Xuanye Zhang, Lina He, Bei Zhang, Yixin Zhou, Shaodong Hong

**Affiliations:** 1 State Key Laboratory of Oncology in South China, Guangzhou, Guangdong, China; 2 Collaborative Innovation Center for Cancer Medicine, Guangzhou, Guangdong, China; 3 Department of VIP Region, Sun Yat-sen University Cancer Center, Guangzhou, Guangdong, China; 4 Guangdong Provincial Key Laboratory of Malignant Tumor Epigenetics and Gene Regulation, Pathology Department, Sun Yat-Sen Memorial Hospital, Guangzhou, Guangdong, China; 5 Department of Radiotherapy, Sun Yat-sen University Cancer Center, Guangzhou, China; 6 Department of Medical Oncology, Sun Yat-sen University Cancer Center, Guangzhou, Guangdong, China

**Keywords:** immunotherapy, lung neoplasms

## Abstract

Anti-PD-1)/programmed cell death-ligand 1 (PD-L1) antibody plus platinum-based chemotherapy (PBC) has replaced PBC as first-line treatment for patients with non-squamous (sq) non-small cell lung cancer (NSCLC) lacking targetable driver mutations. However, few studies have directly compared immune checkpoint inhibitor (ICI) plus chemotherapy with bevacizumab plus chemotherapy (beva +chemo) in this setting. Herein, we conducted an indirect comparison for anti-PD-1/PD-L1 antibody plus chemotherapy (ICI +chemo) versus beva +chemo in non-sq NSCLC using the frequentist methods. The main outcomes analyzed include progression-free survival (PFS), overall survival (OS), and objective response rate (ORR). Data were subtracted from randomized trials comparing ICI +chemo or beva +chemo against PBC. Fourteen trials involving 6165 patients were included. Direct meta-analyses showed that both ICI +chemo (PFS: HR 0.58, OS: HR 0.73, ORR: relative risk (RR) 1.66) and beva +chemo (PFS: HR 0.74, OS: HR 0.89, ORR: RR 1.62) improved clinical outcomes compared with PBC. Indirect comparison showed that ICI +chemo reduced the risk of disease progression (HR 0.78, 95% CI 0.60 to 1.00) and death (HR 0.82, 95% CI 0.71 to 0.94) compared with beva +chemo. The PFS benefits with ICI +chemo over beva +chemo were non-significant in those with negative PD-L1 expression and non-smokers. In conclusion, ICI +chemo is superior to beva +chemo in first-line treatment for non-sq NSCLC.

## Introduction

Lung cancer is the leading cause of cancer-related mortality worldwide.^1^ For decades, platinum-based chemotherapy (PBC) had been the standard-of-care first-line treatment for patients with advanced non-squamous (sq) non-small cell lung cancer (NSCLC) before the era of immune checkpoint inhibitor (ICI) therapies. The addition of bevacizumab to PBC (bevacizumab plus chemotherapy (beva +chemo)) further improved patients’ survival relative to PBC in non-sq NSCLC.^2–4^ For patients lacking sensitizing mutations, the combination of antiprogrammed cell death 1 (PD-1) or antiprogrammed cell death-ligand 1 (PD-L1) antibody with PBC (ICI +chemo) has significantly prolonged overall survival (OS) and progression-free survival (PFS) compared with PBC alone.^5^ However, since the control group of these trials was PBC alone rather than beva +chemo, it remains unknown whether ICI +chemo is superior to beva +chemo for non-sq NSCLC or whether we should retain ICI as second-line treatment following beva +chemo.

Indirect treatment comparison has been demonstrated to provide useful evidence in the absence of randomized controlled trials involving a direct comparison.^6^ Due to the lack of direct comparison, we conducted this indirect meta-analysis to investigate the magnitude of treatment benefit of ICI+chemo over beva +chemo in non-sq NSCLC.

## Methods

### Data sources and searches

PubMed, Embase, Cochrane Library, and major oncology conferences were searched for relevant studies. We used main subject terms including PD-1, PD-L1, bevacizumab, non-small cell lung carcinoma, and randomized controlled trials, etc (online supplemental additional methods).

10.1136/jitc-2021-003431.supp1 Supplementary data

### Data extraction

The following outcomes were extracted from the included trial: PFS, OS, objective response rate (ORR) and treatment-related adverse events (AEs). Other details such as the acronym of the trial, treatment, and patient characteristics were also included in the information sheet.

### Assessment of study quality

Trial quality was assessed by using Cochrane Risk of Bias Tool.^7^

### Statistical analysis

We first performed direct meta-analyses comparing ICI+chemo with chemotherapy, and beva+chemo with chemotherapy, respectively. We calculated the pooled HR for PFS and OS by applying the generic inverse-variance methods model and the pooled relative risks (RRs) for ORR and AEs by using the Mantel-Haenszel method. Heterogeneity was evaluated using Cochran’s Q test; a p value of <0.1 and *I*^2^ of >50% represented statistical heterogeneity, and a random effect model was used; otherwise, a fixed effect model was used.

Linked by arm C (chemotherapy), indirect comparisons between arm A (ICI+chemo) and arm B (beva +chemo) were further performed, applying the frequentist methods with the following formula^8^: log HR_AB_=log HR_AC_−log HR_BC_, and its SE for the log HR was $SE\left(logHR\text{AB}\right)=\sqrt{SE{\left(logHR\text{AC}\right)}^{2}+SE{\left(logHR\text{BC}\right)}^{2}}$. RR was calculated in the same way.

All statistical analyses were conducted using Stata software V.16.0. A two-sided p value of <0.05 defined statistical significance.

## Results

### Eligible studies and patient characteristics

A total of 14 studies were included (online supplemental additional figure S1), 6 of which investigated the efficacy of beva +chemo (n=1264) versus chemotherapy (n=1219), while the other 8 trials explored ICI+chemo (n=2177) versus chemotherapy (n=1505). Detailed characteristics of the included trials are summarized in table 1 and online supplemental additional table S1.

10.1136/jitc-2021-003431.supp2 Supplementary data

10.1136/jitc-2021-003431.supp3 Supplementary data

**Figure 1:**
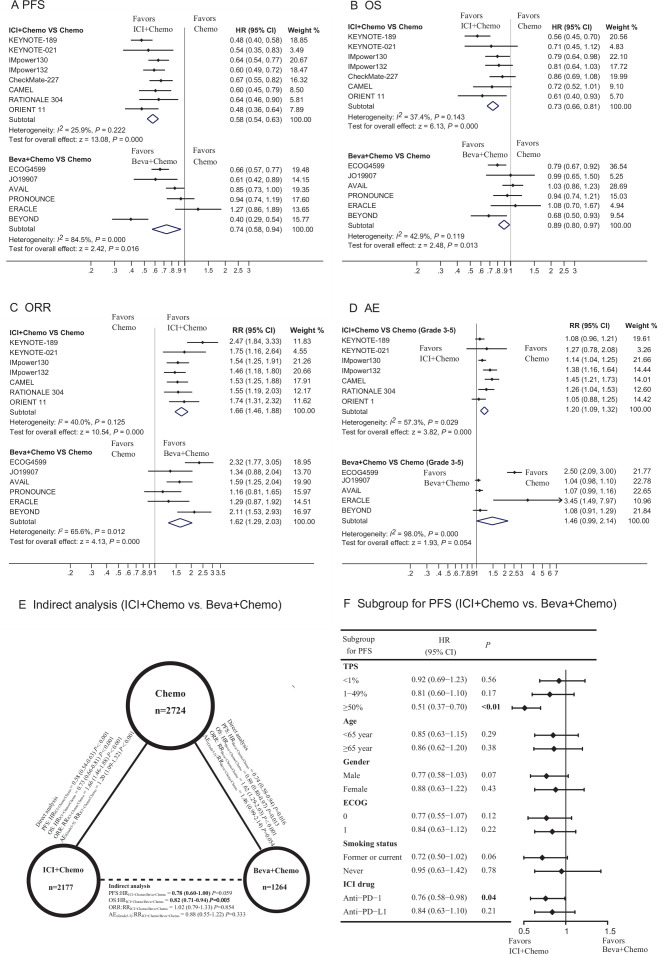
Direct and indirect comparisons among ICI+chemo, beva +chemo and chemotherapy, and subgroup analyses for PFS between ICI+chemo and beva +chemo. (A–D) Forest plot of HR and RR directly comparing PFS, OS, ORR and AE between ICI+chemo or beva +chemo with chemotherapy. The horizontal line crossing the square represents the 95% CI. (E) Solid lines represent the existence of direct comparisons between the treatments, whereas dashed line represents the indirect comparison between ICI+chemo versus beva +chemo. The size of the circle corresponds to the number of enrolled patients. (F) Forest plot of HR indirectly comparing PFS according to patient characteristics at baseline. All statistical tests were two-sided. AE, adverse event; beva, bevacizumab; chemo, chemotherapy; ECOG, Eastern Cooperative Oncology Group; ICI, immune checkpoint inhibitor; ORR, objective response rate; OS, overall survival; PFS, progression-free survival; RR, risk ratio; PD-1, programmed cell death 1; PD-L1, programmed cell death-ligand 1; TPS, tumor proportion score.

**Table 1:** Characteristics of included trials

Trial name*	Arm	N	Age	Male (%)	ECOG 1 (%)	Smoke (%)	ORR (%)	OS (months)	PFS (months)	DOR (months)	HR PFS (95% CI)	HR OS (95% CI)
ECOG4599, 2006	TC +bevacizumab	417	56.0	50.4	60.0	NR	35.0	12.3	6.2	NR	0.66 (0.57 to 0.77)	0.79 (0.67 to 0.92)
	TC	433	58.0	58.4	60.0	NR	15.0	10.3	4.5	NR	Ref	Ref
JO19907, 2010	TC +bevacizumab	121	61.0	63.6	49.0	69.0	60.7	22.8	6.9	6.9	0.61 (0.42 to 0.89)	0.99 (0.65 to 1.50)
	TC	59	60.0	64.4	51.0	68.0	31.0	23.4	5.9	5.6	Ref	Ref
AVAiL, 2010	GP +bevacizumab	351	59.0	62.4	59.0	NR	34.6	13.4	6.5	6.1	0.85 (0.73 to 1.00)	1.03 (0.86 to 1.23)
	GP	347	59.0	64.3	59.0	NR	21.6	13.1	6.1	4.7	Ref	Ref
PRONOUNCE, 2015	AC	182	66.0	57.5	52.7	90.1	23.6	10.5	4.4	NR	1.06 (0.84 to 1.35)	1.07 (0.83 to 1.36)
	TC +bevacizumab	179	65.0	58.1	53.1	96.1	27.4	11.7	5.5	NR	Ref	Ref
ERACLE, 2015	AP	60	60.0	70.0	22.0	70.0	40.0	14.0	8.1	NR	0.79 (0.53 to 1.17)	0.93 (0.60 to 1.42)
	TC +bevacizumab	58	62.0	77.6	21.0	60.0	51.7	14.4	8.3	NR	Ref	Ref
BEYOND, 2015	TC +bevacizumab	138	57.0	54.3	75.0	50.0	54.0	24.3	9.2	8.0	0.40 (0.29 to 0.54)	0.68 (0.50 to 0.93)
	TC	138	56.0	55.8	80.0	44.0	26.0	17.7	6.5	5.3	Ref	Ref
KEYNOTE-189, 2020	AC/P+pembrolizumab	410	65.0	62.0	53.9	88.3	48.0	22.0	9.0	11.2	0.48 (0.40 to 0.58)	0.56 (0.45 to 0.70)
	AC/P	206	63.5	52.9	60.7	87.9	19.4	10.7	4.9	7.8	Ref	Ref
KEYNOTE-021, 2020	AC+pembrolizumab	60	62.5	37.0	58.0	75.0	58.0	34.5	24.5	36.3	0.54 (0.35 to 0.83)	0.71 (0.45 to 1.12)
	AC	63	63.2	41.0	54.0	86.0	33.0	21.1	9.9	22.8	Ref	Ref
IMpower130, 2019	Nab-TC +atezolizumab	451	64.0	59.0	58.0	89.0	49.2	18.6	7.0	8.4	0.64 (0.54 to 0.77)	0.79 (0.64 to 0.98)
	Nab-TC	228	65.0	59.0	60.0	92.0	31.9	13.9	5.5	6.1	Ref	Ref
IMpower132, 2020	AC/p+atezolizumab	292	64.0	65.8	56.8	NR	47.0	18.1	7.6	10.1	0.60 (0.49 to 0.72)	0.81 (0.64 to 1.03)
	AC/P	286	63.0	67.1	59.9	NR	32.0	13.6	5.2	7.2	Ref	Ref
CheckMate 227, 2020	AC/P+nivolumab	270	63.0	36.0	61.0	82.0	NR	18.8	8.7	NR	0.67 (0.55 to 0.82)	0.86 (0.69 to 1.08)
	AC/P	273	63.0	37.0	69.0	75.0	NR	15.5	5.8	6.2	Ref	Ref
CAMEL, 2019	AC+camrelizumab	205	59.0	71.2	76.6	NR	60.0	NR	11.3	17.6	0.60 (0.45 to 0.79)	0.72 (0.52 to 1.01)
	AC	207	61.0	72.0	82.5	NR	39.1	20.9	8.3	9.9	Ref	Ref
RATIONALE 304, 2020	AC/P+tislelizumab	223	60.0	75.3	75.8	65.9	57.4	NR	9.7	8.5	0.65 (0.46 to 0.90)	NR
	AC/P	111	61.0	71.2	78.4	59.5	36.9	NR	7.6	6.0	Ref	NR
ORIENT 11, 2020	AC/P+sintilimab	266	61.0	76.7	71.4	64.3	51.9	NR	8.9	NR	0.48 (0.36 to 0.64)	0.61 (0.40 to 0.93)
	AC/P	131	61.0	75.6	74.0	66.4	29.8	NR	5.0	5.5	Ref	Ref

*The references for the enrolled trials are listed in online supplemental additional table S1.

AC, pemetrexed +carboplatin; AC/P, pemetrexed +carboplatin/cisplatin; AP, pemetrexed +cisplatin; CI, confidence interval; DOR, duration of response; ECOG1, Eastern Cooperative Oncology Group 1; GP, gemcitabine +cisplatin; Nab-TC, nanoparticle albumin-bound paclitaxel +carboplatin; NR, not report; ORR, objective response rate; OS, overall survival; PFS, progression-free survival; Ref, reference; TC, paclitaxel +carboplatin.

### Direct comparisons between ICI+Chemo or Beva+Chemo and chemotherapy

The pooled results showed that ICI+chemo led to significant improvements in PFS (HR _ICI+chemo/chemo_ 0.58, 95% CI 0.54 to 0.63), OS (HR_ICI+chemo/chemo_ 0.73, 95% CI 0.66 to 0.81), and ORR (RR_ICI+chemo/chemo_ 1.66, 95% CI 1.46 to 1.88) compared with PBC. Likewise, treatment benefits were found with the addition of bevacizumab to PBC in terms of PFS (HR_beva+chemo/chemo_ 0.74, 95% CI 0.58 to 0.94), OS (HR_beva+chemo/chemo_ 0.89, 95% CI 0.80 to 0.97), and ORR (RR_beva+chemo/chemo_ 1.62, 95% CI 1.29 to 2.03) (figure 1A–C). Nevertheless, the two combinatorial treatments increased the risk of ≥grade 3 AEs (RR_ICI+chemo/chemo_ 1.20, 95% CI 1.09 to 1.32; RR_beva+chemo/chemo_ 1.46, 0.99 to 2.14; figure 1D).

### Indirect comparisons between ICI+Chemo and Beva+Chemo

In indirect analyses, ICI+chemo has a trend to reduce the risk of disease progression or death (HR_ICI+chemo/beva+chemo_ 0.78, 95% CI 0.60 to 1.00; p=0.059) compared with beva +chemo, and is superior to beva +chemo in reducing the risk of death (HR _CI+chemo/beva+chemo_ 0.82, 95% CI 0.71 to 0.94; p<0.01) (figure 1E). However, the two regimens were similar in terms of ORR (RR_ICI+chemo/beva+chemo_ 1.02, 95% CI 0.79 to 1.33; p=0.85) (figure 1E).

In subgroup analyses by PD-L1 expression level, when compared with beva +chemo, ICI+chemo led to a significantly longer PFS for patients with PD-L1 tumor proportion score (TPS) of ≥50% (HR_ICI+chemo/beva+chemo_ 0.51, 95% CI 0.37 to 0.70; p<0.01) but not for patients with PD-L1 TPS of 1%–49% (HR_ICI+chemo/beva+chemo_ 0.81, 95% CI 0.60 to 1.10; p=0.17), or PD-L1 TPS of <1% (HR_ICI+chemo/beva+chemo_ 0.92, 95% CI 0.69 to 1.23; p=0.56) (figure 1F). In most of the other subgroups, there was a consistent trend towards improved PFS with ICI+chemo versus beva +chemo, except that in non-smokers, the HR for PFS was near 1 (0.95, 95% CI 0.63 to 1.42).

For safety profiles, the frequency of grade 3 or more severe AEs was similar between ICI+chemo and beva +chemo (RR_ICI+chemo/beva+chemo_ 0.82, 95% CI 0.55 to 1.22; p=0.33). However, treatment-related deaths occurred less for those receiving ICI+chemo than for those treated with beva +chemo (RR_ICI+chemo/beva+chemo_ 0.56, 95% CI 0.32 to 0.97; p=0.02) (online supplemental additional figure S2).

10.1136/jitc-2021-003431.supp4 Supplementary data

## Discussion

In this indirect meta-analysis, ICI +chemo was found to prolong both PFS and OS without increasing toxicity when compared with beva +chemo in the first-line treatment for advanced non-sq NSCLC. The PFS benefit was more obvious in patients with PD-L1 TPS of ≥50%. These findings consolidate the role of ICI in front-line treatment of patients with NSCLC, especially for those with high PD-L1 expression.

In updated analysis from IMpower150 study, atezolizumab plus carboplatin plus paclitaxel failed to prolong PFS (HR 0.91, 95% CI 0.78 to 1.06) or OS (HR 0.85, 95% CI 0.71 to 1.03) compared with bevacizumab plus carboplatin plus paclitaxel.^9^ This raised growing concern about whether ICI should be placed in first-line setting. This concern is relevant because few studies have used beva+chemo as control arm despite the fact that this regimen is more efficacious than chemotherapy alone. In our study with more patients analyzed, ICI +chemo and beva +chemo yielded similar ORR (RR 1.02, 95% CI 0.79 to 1.33). However, ICI +chemo was associated with a 22% reduction in the risk of disease progression or death (HR 0.78, 95% CI 0.60 to 1.00) and a 18% reduction in the risk of death (HR 0.82, 95% CI 0.71 to 0.94) compared with beva +chemo. One important reason for the discrepancies among ORR, PFS and OS benefit was the longer duration of response for patients treated with ICI +chemo than with beva +chemo (median 8.4–36.3 months vs 6.1–8.0 months, table 1). Another important finding was that the magnitude of survival benefit with ICI +chemo was reduced when the control group shifted from chemotherapy to beva +chemo (our previous pooled analysis showed that ICI +chemo was associated with 38% and 32% reduction in the risk of disease progression/death and death compared with chemotherapy alone, respectively^5^). This implied that the delayed application of bevacizumab in most ICI +chemo trials may be detrimental for patients in the control group. One open question is whether combing ICI, bevacizumab and chemotherapy together in a first-line setting will further improve survival benefit. This is partially addressed in the IMpower150^9^ and LUN 17-139^10^ studies, both of which showed that ICI plus beva+chemo (ICI +beva+chemo) prolongs PFS compared with beva +chemo, but at the expense of more toxicities. However, whether ICI +beva+chemo outperforms ICI +chemo remains a question to be answered with randomized studies in the future. Interestingly, subgroup analysis from IMpower150 indicates that ICI +beva+chemo may provide survival benefit in patients who are less likely to respond to ICI, such as those with liver metastasis or epidermal growth factor receptor (EGFR) mutation.^9^

The PD*-*L1 expression was an established biomarker for anti-PD-1/PD-L1 monotherapy in NSCLC and remains a suitable biomarker to predict the PFS benefit with ICI+chemo versus beva +chemo in this study. PD-L1 TPS of ≥50% was associated with significantly longer PFS (HR 0.51, 95% CI 0.37 to 0.70) with ICI+chemo versus beva +chemo, while patients with PD-L1 of less than 1% had comparable PFS when treated with ICI+chemo or beva +chemo (HR 0.92, 95% CI 0.69 to 1.23). ICI+chemo also did not produce PFS benefit in non-smokers. Further studies were warranted to explore predictive biomarker to differentiate beneficiary from ICI+chemo versus beva +chemo.

In terms of toxicity, ICI +chemo and beva +chemo were comparable for AEs of ≥grade 3 (HR 0.82, 95% CI 0.55 to 1.22), but the risk of AEs leading to death was significantly lower with ICI +chemo versus beva +chemo (HR 0.56, 95% CI 0.32 to 0.97). However, since the profiles of AEs for ICI and bevacizumab were different, the risk of AEs should be assessed individually. For example, patients with hypertension or high bleeding risk might suffer greater risk from bevacizumab, while patients with autoimmune disease might suffer greater risk from ICI.^11^

Based on our observation, we cautiously postulate the following recommendations: for patients with PD-L1 TPS of at least 50% and without contraindications for immunotherapy, ICI+chemo should be preferred compared with beva+chemo; for patients with PD-L1 TPS of less than 50%, ICI+chemo is recommended, but beva+chemo could serve as an alternative, especially for those with PD-L1 TPS of less than 1% or/and with high risk of developing immune-related AEs or hyperprogression disease.^11^

The high quality of the enrolled trials and the low heterogeneity between trials make this analysis reliable. Nevertheless, several limitations should be noted. First, this is an indirect analysis and due to the different inclusion and exclusion criteria between the ICI+chemo trials and beva+chemo trials, the patients’ characteristics might not be well balanced between the two groups. Thus, the result should be interpreted with extra caution. However, considering that a prospective trial that compares ICI+chemo with beva+chemo is unlikely to be conducted, this analysis would meet current clinical needs. Second, the results regarding OS should be further investigated in prospective trials, since patients who received beva+chemo in this study did not cross over to immunotherapy in later-line treatments.

In conclusion, ICI+chemo was associated with significantly longer PFS and OS and comparable risk of AEs compared with beva +chemo. PD-L1 expression might be a predictive biomarker of PFS benefit with ICI+chemo versus beva +chemo.
