# Supplementary material for: PD-1/PD-L1 inhibitor plus chemotherapy versus bevacizumab plus chemotherapy in first-line treatment for non-squamous non-small-cell lung cancer
Source: J Immunother Cancer. 2021 Nov 8;9(11):e003431. doi: 10.1136/jitc-2021-003431 (PMC8576478; doi:10.1136/jitc-2021-003431)
Supplement: Supplementary data [file jitc-2021-003431supp001.pdf]

Additional file 1 Supplemental Methods

Search strategies for PubMed, EMBASE, and Cochrane database

Pubmed:1040 results (2000-2020)

(("pembrolizumab" [Supplementary Concept] OR "lambrolizumab" [Title/Abstract] OR "Keytruda" [Title/Abstract] OR "MK-3475" [Title/Abstract] OR "nivolumab" [Supplementary Concept] OR "MDX-1106" [Title/Abstract] OR "ONO-4538" [Title/Abstract] OR "BMS-936558" [Title/Abstract] OR "Opdivo"[Title/Abstract] OR "atezolizumab"[Supplementary Concept] OR "MPDL3280A"[Title/Abstract] OR "Tecentriq"[Title/Abstract] OR"RG7446"[Title/Abstract] OR "RG-7446"[Title/Abstract] OR "Durvalumab" [Title/Abstract] OR "Imfinzi" [Title/Abstract] OR " MEDI4736" [Title/Abstract] OR "Camrelizumab" [Title/Abstract] OR "SHR-1210" [Title/Abstract] OR " Tislelizumab" [Title/Abstract] OR "Sintilimab" [Title/Abstract] OR " IBI 308" [Title/Abstract] OR "anti-PDL1"[Title/Abstract] OR "anti-PD1"[Title/Abstract] OR "PD-1"[Title/Abstract] OR "PD-L1"[Title/Abstract]OR "Programmed Death 1"[Title/Abstract] OR "Programmed Cell Death 1 Receptor"[Title/Abstract] OR "Programmed Death-Ligand 1"[Title/Abstract] OR "programmed cell death 1 ligand 1 protein"[Title/Abstract] OR "immune checkpoint inhibitor"[Title/Abstract] OR "immune therapy"[Title/Abstract] OR "immunotherapy"[Title/Abstract])) OR (("Bevacizumab"[Title/Abstract] OR "Avastin"[Title/Abstract])) AND ((((("non-squamous"[Title/Abstract]) AND "lung cancer"[Title/Abstract]) AND (((((NSCLC[Title/Abstract]) OR "Non Small Cell"[Title/Abstract]) OR "Non-Small-Cell"[Title/Abstract]) OR "Non-Small Cell"[Title/Abstract]))) OR "Carcinoma, Non-Small-Cell Lung"[Mesh]) AND (("clinical trials as topic "[MeSH Terms] OR "Randomized clinical trial"[Title/Abstract] OR "phase"[Title/Abstract]))

Embase:1238 results (2000-2020)

(‘pembrolizumab’/exp OR ‘lambrolizumab’:ab,ti OR ‘Keytruda’:ab,ti OR ‘MK-3475’:ab,ti OR ‘nivolumab’/exp OR ‘MDX-1106’:ab,ti OR ‘ONO-4538’:ab,ti OR ‘BMS-936558’:ab,ti OR ‘Opdivo’:ab,ti OR ‘atezolizumab’/exp OR ‘MPDL3280A’:ab,ti OR ‘Tecentriq’:ab,ti OR ‘RG7446’:ab,ti OR ‘RG-7446’:ab,ti OR ‘Durvalumab’:ab,ti OR ‘Imfinzi’:ab,ti OR ‘MEDI4736’:ab,ti OR ‘Camrelizumab’/exp OR ‘SHR-1210’:ab,ti OR ‘Tislelizumab’/exp OR ‘Sintilimab’/exp OR ‘IBI 308’:ab,ti OR ‘anti-PDL1’:ab,ti OR ‘anti-PD1’:ab,ti OR ‘PD-1’:ab,ti OR ‘PD-L1’ab,ti OR ‘Programmed Death 1’ab,ti OR ‘Programmed Cell Death 1 Receptor’:ab,ti OR ‘Programmed Death-Ligand 1’:ab,ti OR ‘programmed cell death 1 ligand 1 protein’:ab,ti OR ‘immune checkpoint inhibitor’:ab,ti OR ‘immune therapy’:ab,ti OR ‘immunotherapy’:ab,ti) OR (‘Bevacizumab’/exp OR ‘Avastin’:ab,ti) AND (('non small cell lung cancer'/exp OR ('lung':ab,ti AND ('NSCLC':ab,ti OR 'non small cell':ab,ti OR 'non-small-cell':ab,ti OR 'non-small cell':ab,ti)) AND (‘randomized controlled trial’/exp)

Cochrane:699 results (699 trials) (2000-2020)

#1 MeSH descriptor: [Carcinoma, Non-Small-Cell Lung] explode all trees

#2 ‘lung’ AND ("Non Small Cell" OR "Non-Small Cell" OR "Non-Small-Cell") OR "NSCLC"

#3 non-squamous: ti, ab, kw

#4 (#1OR #2) AND #3

#5 (pembrolizumab OR lambrolizumab OR Keytruda OR MK-3475 OR nivolumab OR MDX-1106 OR ONO-4538 OR BMS-936558 OR Opdivo OR atezolizumab OR MPDL3280A OR Tecentriq OR RG7446 OR RG-7446 OR Durvalumab OR Imfinzi OR MEDI4736 OR Camrelizumab OR SHR-1210 OR Tislelizumab OR Sintilimab OR 'IBI 308' OR 'anti-PDL1' OR 'anti-PD1' OR 'PD-1' OR PD-L1 OR 'Programmed Death 1' OR ‘Programmed Cell Death 1 Receptor' OR 'Programmed Death-Ligand 1' OR ‘programmed cell death 1 ligand 1 protein' OR ‘immune checkpoint inhibitor’ OR 'immune therapy’ OR immunotherapy): ti, ab, kw

#6 (Bevacizumab OR Avastin): ti, ab, kw

#7 #5 OR #6

#8 #4 AND #7
