# Supplementary figures and images for: PD-1/PD-L1 inhibitor plus chemotherapy versus bevacizumab plus chemotherapy in first-line treatment for non-squamous non-small-cell lung cancer
Source: J Immunother Cancer. 2021 Nov 8;9(11):e003431. doi: 10.1136/jitc-2021-003431 (PMC8576478; doi:10.1136/jitc-2021-003431)

**Additional file 2 Figure S1**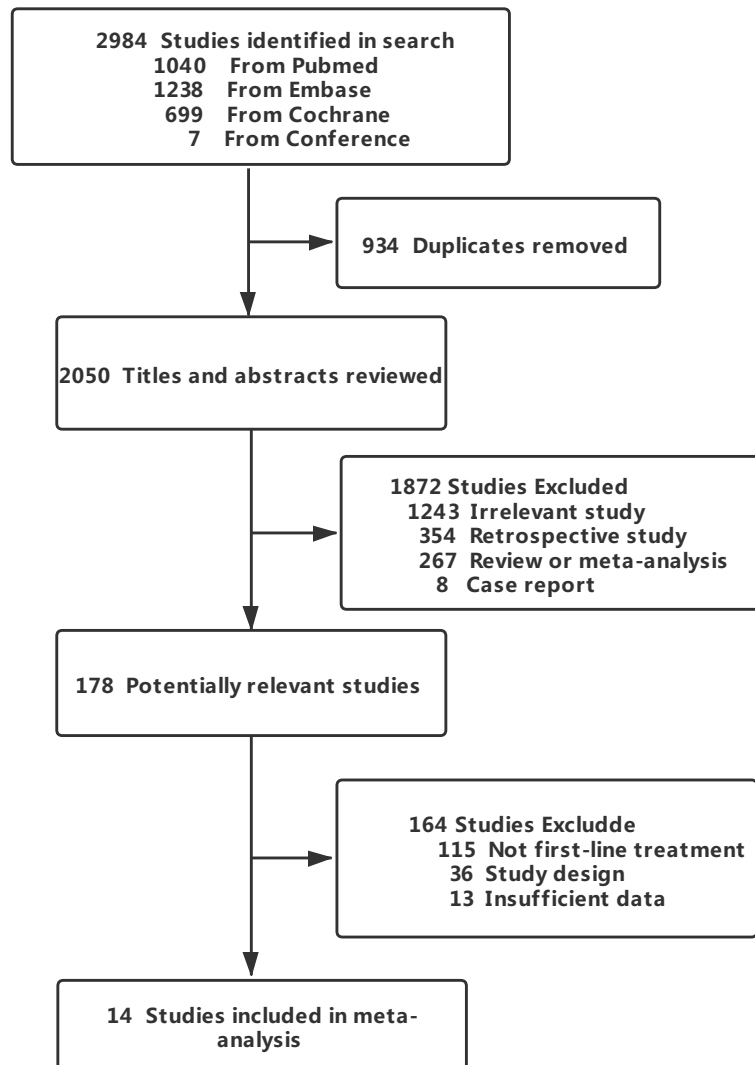

Supplement: Supplementary data [file jitc-2021-003431supp002.pdf]

Additional file 4 Figure S2

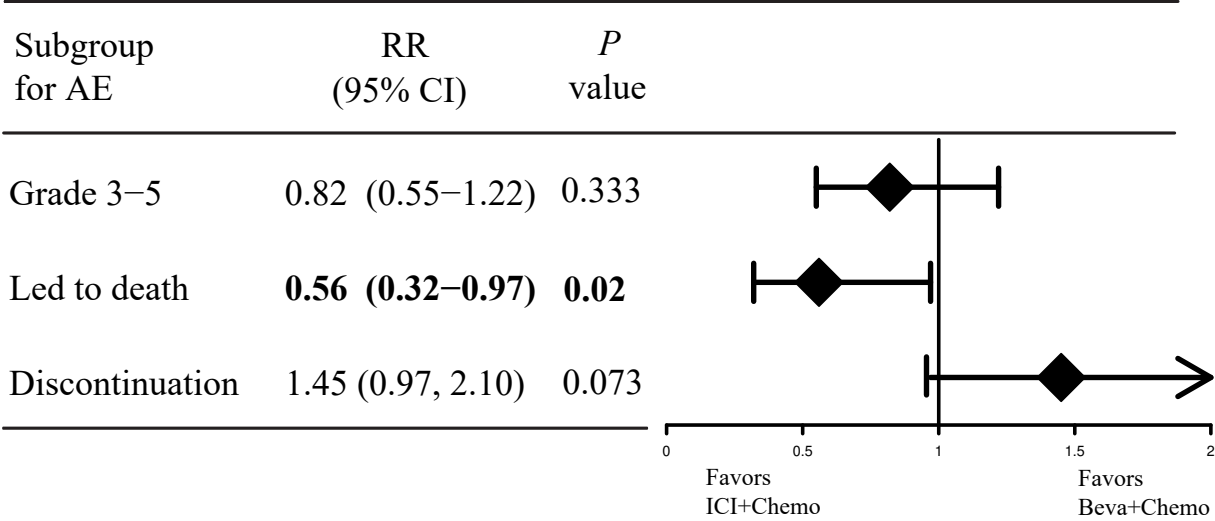

Supplement: Supplementary data [file jitc-2021-003431supp004.pdf]
