# Supplementary material for: PD-1/PD-L1 inhibitor plus chemotherapy versus bevacizumab plus chemotherapy in first-line treatment for non-squamous non-small-cell lung cancer
Source: J Immunother Cancer. 2021 Nov 8;9(11):e003431. doi: 10.1136/jitc-2021-003431 (PMC8576478; doi:10.1136/jitc-2021-003431)
Supplement: Supplementary data [file jitc-2021-003431supp003.pdf]

Additional file 3 Table S1. Quality assessment by Cochrane Collaboration's tool.

Trial	Sequence generation	Allocation concealment	Blinding	Incomplete outcome data	Selective reporting	Other source of bias
ECOG4599[1]	Adequate	Not clear	Adequate[a]	Adequate	Adequate	
JO19907[2]	Adequate	Not clear	Adequate[a]	Adequate	Adequate	
AVAiL[3]	Adequate	Adequate (Central allocation)	Adequate[a]	Adequate	Adequate	
PRONOUNCE[4]	Adequate	Not clear	Adequate[a]	Adequate	Adequate	
ERACLE[5]	Adequate	Adequate (Central allocation)	Adequate[a]	Adequate	Adequate	
BEYOND[6]	Adequate	Adequate (Central allocation)	Adequate[a]	Adequate	Adequate	
KEYNOTE-189[7]	Adequate	Adequate (Central allocation)	Adequate[a]	Adequate	Adequate	
KEYNOTE-021[8]	Adequate	Adequate (Central allocation)	Adequate[a]	Adequate	Adequate	
IMpower130[9]	Adequate	Adequate (Central allocation)	Adequate[a]	Adequate	Adequate	
IMpower132[10]	Adequate	Not clear	Adequate[a]	Adequate	Adequate	
CheckMtae 227[11]	Adequate	Adequate (Central allocation)	Adequate[a]	Adequate	Adequate	
CAMEL[12]	Adequate	Not clear	Adequate[a]	Adequate	Adequate	
RATIONALE 304[13]	Adequate	Adequate (Central allocation)	Adequate[a]	Inadequate[b]	Adequate	
ORIENT 11[14]	Adequate	Adequate (Central allocation)	Adequate[a]	Adequate	Adequate	

a: The sponsor, investigator and subject were aware of the treatment administration but the response to treatment was assessed by means of blinded, independent, central radiologic review.

b: Absence of data of OS.

Reference

1. Sandler A, Gray R, Perry M, Brahmer J, Schiller J, Dowlati A, Lilenbaum R, Johnson D: **Paclitaxel-carboplatin alone or with bevacizumab for non-small-cell lung cancer**. *The New England journal of medicine* 2006, **355**(24):2542-2550.
2. Niho S, Kunitoh H, Nokihara H, Horai T, Ichinose Y, Hida T, Yamamoto N, Kawahara M, Shinkai T, Nakagawa K *et al*: **Randomized phase II study of first-line carboplatin-paclitaxel with or without bevacizumab in Japanese patients with advanced non-squamous non-small-cell lung cancer**. *Lung cancer (Amsterdam, Netherlands)* 2012, **76**(3):362-367.
3. Reck M, von Pawel J, Zatloukal P, Ramlau R, Gorbounova V, Hirsh V, Leighl N, Mezger J, Archer V, Moore N *et al*: **Phase III trial of cisplatin plus gemcitabine with either placebo or bevacizumab as first-line therapy for nonsquamous non-small-cell lung cancer: AVAil**. *Journal of clinical oncology : official journal of the American Society of Clinical Oncology* 2009, **27**(8):1227-1234.
4. Zinner R, Obasaju C, Spigel D, Weaver R, Beck J, Waterhouse D, Modiano M, Hrinczenko B, Nikolinakos P, Liu J *et al*: **PRONOUNCE: randomized, open-label, phase III study of first-line pemetrexed + carboplatin followed by maintenance pemetrexed versus paclitaxel + carboplatin + bevacizumab followed by maintenance bevacizumab in patients ith advanced nonsquamous non-small-cell lung cancer**. *Journal of thoracic oncology : official publication of the*

International Association for the Study of Lung Cancer 2015, **10**(1):134-142.

5. Galetta D, Cinieri S, Pisconti S, Gebbia V, Morabito A, Borsellino N, Maiello E, Febbraro A, Catino A, Rizzo P *et al*: **Cisplatin/Pemetrexed Followed by Maintenance Pemetrexed Versus Carboplatin/Paclitaxel/Bevacizumab Followed by Maintenance Bevacizumab in Advanced Nonsquamous Lung Cancer: The GOIM (Gruppo Oncologico Italia Meridionale) ERACLE Phase III Randomized Trial**. *Clinical lung cancer* 2015, **16**(4):262-273.
6. Zhou C, Wu Y, Chen G, Liu X, Zhu Y, Lu S, Feng J, He J, Han B, Wang J *et al*: **BEYOND: A Randomized, Double-Blind, Placebo-Controlled, Multicenter, Phase III Study of First-Line Carboplatin/Paclitaxel Plus Bevacizumab or Placebo in Chinese Patients With Advanced or Recurrent Nonsquamous Non-Small-Cell Lung Cancer**. *Journal of clinical oncology : official journal of the American Society of Clinical Oncology* 2015, **33**(19):2197-2204.
7. Gandhi L, Rodríguez-Abreu D, Gadgeel S, Esteban E, Felip E, De Angelis F, Domine M, Clingan P, Hochmair M, Powell S *et al*: **Pembrolizumab plus Chemotherapy in Metastatic Non-Small-Cell Lung Cancer**. *The New England journal of medicine* 2018, **378**(22):2078-2092.
8. Langer C, Gadgeel S, Borghaei H, Papadimitrakopoulou V, Patnaik A, Powell S, Gentzler R, Martins R, Stevenson J, Jalal S *et al*: **Carboplatin and pemetrexed with or without pembrolizumab for advanced, non-squamous non-small-cell lung cancer: a randomised, phase 2 cohort of the open-label KEYNOTE-021 study**. *The Lancet Oncology* 2016, **17**(11):1497-1508.
9. West H, McCleod M, Hussein M, Morabito A, Rittmeyer A, Conter H, Kopp H, Daniel D, McCune S, Mekhail T *et al*: **Atezolizumab in combination with carboplatin plus nab-paclitaxel chemotherapy compared with chemotherapy alone as first-line treatment for metastatic non-squamous non-small-cell lung cancer (IMpower130): a multicentre, randomised, open-label, phase 3 trial**. *The Lancet Oncology* 2019, **20**(7):924-937.
10. Nishio M, Barlesi F, West H, Ball S, Bordoni R, Cobo M, Longeras PD, Goldschmidt J, Jr., Novello S, Orlandi F *et al*: **Atezolizumab Plus Chemotherapy for First-Line Treatment of Nonsquamous NSCLC: Results From the Randomized Phase 3 IMpower132 Trial**. *J Thorac Oncol* 2021, **16**(4):653-664.
11. Hellmann M, Paz-Ares L, Bernabe Caro R, Zurawski B, Kim S, Carcereny Costa E, Park K, Alexandru A, Lupinacci L, de la Mora Jimenez E *et al*: **Nivolumab plus Ipilimumab in Advanced Non-Small-Cell Lung Cancer**. *The New England journal of medicine* 2019, **381**(21):2020-2031.
12. Zhou C, Chen G, Huang Y, Zhou J, Lin L, Feng J, Wang Z, Shu Y, Shi J, Hu Y *et al*: **Camrelizumab plus carboplatin and pemetrexed versus chemotherapy alone in chemotherapy-naive patients with advanced non-squamous non-small-cell lung cancer (CameL): a randomised, open-label, multicentre, phase 3 trial**. *The Lancet Respiratory medicine* 2021, **9**(3):305-314.
13. Lu S, Wang J, Yu Y, Yu X, Hu Y, Ai X, Ma Z, Li X, Zhuang W, Liu Y *et al*: **Tislelizumab Plus Chemotherapy as First-Line Treatment for Locally Advanced or Metastatic Nonsquamous NSCLC (RATIONALE 304): A Randomized Phase 3 Trial**. *Journal of thoracic oncology : official publication of the International Association for the Study of Lung Cancer* 2021.
14. Yang Y, Wang Z, Fang J, Yu Q, Han B, Cang S, Chen G, Mei X, Yang Z, Ma R *et al*: **Efficacy and Safety of Sintilimab Plus Pemetrexed and Platinum as First-Line Treatment for Locally Advanced or Metastatic Nonsquamous NSCLC: a Randomized, Double-Blind, Phase 3 Study (Oncology pRogram by InnovENT anti-PD-1-11)**. *J Thorac Oncol* 2020, **15**(10):1636-1646.
